# Design and fabrication of a passive droplet dispenser for portable high resolution imaging system

**DOI:** 10.1038/srep41482

**Published:** 2017-01-27

**Authors:** Tahseen Kamal, Rachel Watkins, Zijian Cen, Jaden Rubinstein, Gary Kong, Woei Ming Lee

**Affiliations:** 1 Research School of Engineering, College of Engineering and Computer Science, The Australian National University, Canberra, ACT 2601, Australia; 2 Plant Biosecurity Cooperative, Research Centre, LPO Box 5012, Bruce, ACT 2617, Australia; 3 Australia Research Council Centre of Excellence in Advanced Molecular Imaging, Australian National University, Australia

## Abstract

Moldless lens manufacturing techniques using standard droplet dispensing technology often require precise control over pressure to initiate fluid flow and control droplet formation. We have determined a series of interfacial fluid parameters optimised using standard 3D printed tools to extract, dispense and capture a single silicone droplet that is then cured to obtain high quality lenses. The dispensing process relies on the recapitulation of liquid dripping action (Rayleigh-Plateau instability) and the capturing method uses the interplay of gravitational force, capillary forces and liquid pinning to control the droplet shape. The key advantage of the passive lens fabrication approach is rapid scale-up using 3D printing by avoiding complex dispensing tools. We characterise the quality of the lenses fabricated using the passive approach by measuring wavefront aberration and high resolution imaging. The fabricated lenses are then integrated into a portable imaging system; a wearable thimble imaging device with a detachable camera housing, that is constructed for field imaging. This paper provides the full exposition of steps, from lens fabrication to imaging platform, necessary to construct a standalone high resolution imaging system. The simplicity of our methodology can be implemented using a regular desktop 3D printer and commercially available digital imaging systems.

Decentralisation of complex scientific instruments by leveraging on consumer electronics and mobile devices^1^, i.e. laptops, smartphones, mobile smart devices, is becoming useful in a variety of applications ranging from point-of-care medicine^2^, geophysics research^3^,^4^, education^5^ and nature conservation^6^. These vast amount of information from mobile devices are intended to aid researchers or medical practitioners and rapidly tackle multifaceted problems on large scale^7^,^8^ in a cost-effective manner. The combination of digital imaging with new computational tools^9^, biomarkers^10^ and 3D printing^11^ have ushered in practical *in-vivo* imaging screening on mobile devices^12^,^13^,^14^ for primary care and low resources settings. This is especially useful in image-intensive medical practices; ophthalmology, dermatology and pathology, where the accurate characterisation of samples at macroscopic and microscopic level is crucial for identification of diseases. There is now an emerging field in mobile microscopy systems^1^,^2^,^8^,^10^ using compact computing and imaging devices that aims to tackle the needs to decentralise microscopy systems. These microscopy systems can be separated into two categories based on optics, namely; lensless and lens-based. The lensless approach provides greater flexibility in obtaining magnified images over large field of view at the cost of computational time^9^. On the other hand, simple add-on lenses^10^ are limited to fixed aperture sizes and focal lengths, but offer greater ease of use at minimal costs.

Recently, the moldless fabrication of high performance silicone lenses provides rapid access to direct fabrication techniques to obtain low cost but high performance lenses for high resolution microscopy imaging on smartphone devices^15^,^16^,^17^,^18^. While many of these fabrication techniques are simple, they still require accurate dispensing devices, such as a syringe. Droplets are often transient and are susceptible to interaction on interfaces (liquid, air, solid) which makes it difficult to control doing deposition. This is further complicated by instability of satellite droplets forming during liquid jetting process^19^,^20^ which can create unnecessary droplets during curing, which distorts the final droplet shape. The purpose of an active dispensing process is used to control the volume of polymer droplet as it is being deposited and cures, which in doing so retains the shapes of the droplet. In the case of polymer refractive lenses, a parabolic profile of the droplet needs to be held in place as the polymer cross-links. Existing rapid photo-curing process and thermal-based moldless techniques^15^,^16^,^17^,^18^ require precise deposition to stabilise the shape of each droplet as they are deposited on a solid surface. As a result, the moldless technique does not lend itself to a widely adopted lens fabrication technique. Therefore, in principle, a simple passive droplet formation process could lend itself to an accessible lens fabrication tool.

While consumer smartphone systems are rapidly becoming a preferred diagnosis platform due to consumer acceptance, they are often limited by design constraints set by the manufacturers^13^. This meant that smartphone microscopes retain fixed form factors i.e. orientation of imaging sensors and display, that cannot be easily reconfigured. When compared against other flexible imaging devices such as digital endoscope or inspection scopes, the rigid smartphone design offers much less dexterity. As such, a “deconstructed” imaging system, where the camera, computing and display are independent, would be more adaptable to different imaging needs of developer or user.

In this paper, we propose a passive droplet dispensing and capturing approach that can be tailored to fabricate high quality droplet lenses. The droplet lenses is then showed to be integrated with a “wearable” microscopy system and perform field microscopy. In droplet fabrication method, we carefully examine the three steps; extract, dispense and deposit, that is necessary to stably deposit a single droplet onto a curing holder using standard 3D printing technology. In the extraction step, we investigate the use of capillary action to extract a fixed amount of liquid silicone. A fixed volume of silicone liquid is then accurately dispensed onto a droplet holder by carefully adjust capillary and gravitational forces. A holder is designed to stably retain the droplet shape during curing by controlling wetting/pinning. Using 3D printing substrates, we tested a set of solid substrate that optimises the interfacial parameters so as to realise the concept of passive droplet dispenser and harvested high quality optical lenses out of silicone droplets. The silicone lenses are carefully characterised using a Shack Hartmann wavefront sensor and surface profiler. The lenses are then integrated into a standalone imaging system, based on Raspberry Pi that is both cost-efficient small form factor and modular. Standard imaging targets (histology slides, USAF1951) are used to provide a guided assessment of the overall imaging quality and resolution of the system. The flexibility and simplicity of our methodology means that almost the entire system can be fabricated from a regular desktop 3D printer (UP-mini), commercially available materials such as transparent silicone/polydimethylsiloxane (PDMS) and standard hard polymer material (acrylonitrile-butadiene-styrene, ABS). In the following sections, we provide a clear exposition of fabrication techniques starting from production of lenses to housing design (camera, interface, computing unit) and implementing microscopic and macroscopic imaging.

## Harvesting droplet lenses with dripping silicone liquid

Droplet formation is ubiquitous in many facets of droplet dispensing technology where a flowing stream of fluid filament collapses into smaller masses of fluid drops^20^. The shapes of droplets are highly susceptible to interaction at interfaces which is also difficult to control on solid surfaces. A flowing fluid possesses certain amount of instability in terms of velocity, radius of the flowing jet and length^21^. While modulated liquid jets are a result of liquid instability, which has been used to create micro-optical devices^22^ or multifunctional microspheres^23^, they are not desired in moldless fabrication of refractive lenses^15^,^16^,^18^. This is because the formation of satellite liquid droplets changes the final deposited droplet and lead to a distorted parabolic profile i.e. poor lens. In order to avoid the formation of satellite droplets, each droplet needs to be smoothly transferred and cured thereafter. Active dispensing method minimise the formation of satellite droplets by controlling pressure. Since each active dispenser needs to be equipped with mechanical controls, it will be difficult to scale up rapidly. In conventional dispensing tools, a syringe is used to extract a precise amount of liquid by negative pressure which is followed by a positive pressure to dispense liquid through a nozzle of syringe. As the drop grows in size, the weight of the droplet exceeds the surface tension and capillary force where a discrete droplet is formed. For a simple narrow opening, i.e. nozzle, a small precise amount of pressure is usually applied at the distal end of the nozzle to initiate this droplet growing process. This control mechanism is crucial to ensure that a single droplet is formed. Instead of active force, we propose the use of capillary and gravitational forces to initiate the extraction and dispensing of a droplet.

**Figure 1:**
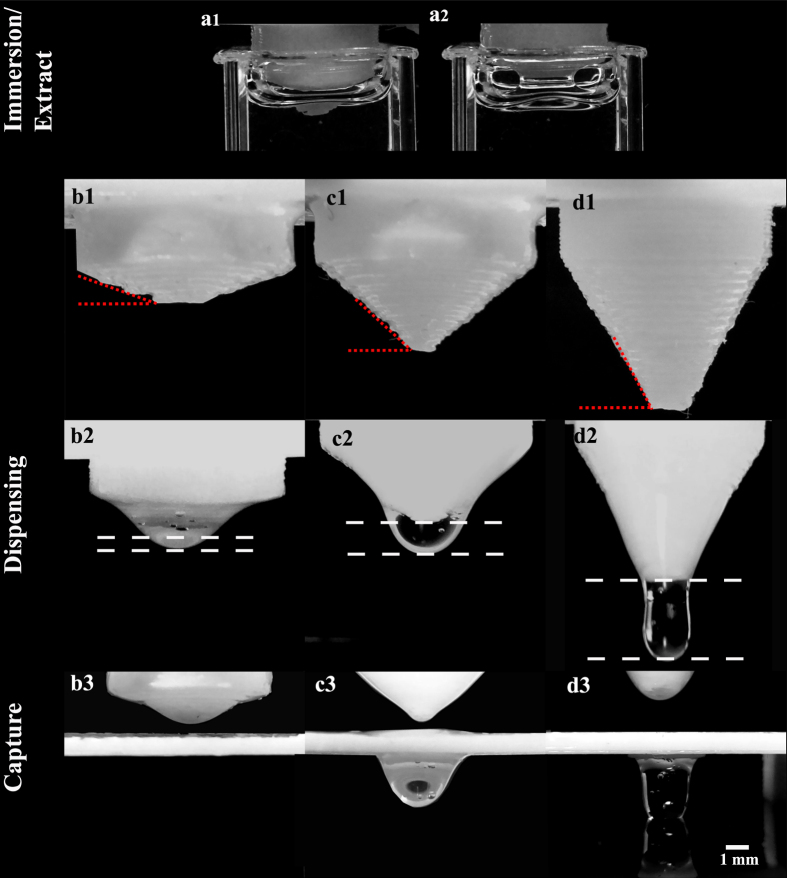
Immersion/extract, dispensing, capturing single silicone droplet using the proposed 3D substrates. (**a1**–**a2**) Shows the immersion process for extracting fixed fluid volume using the conic substrate and a reservoir of fluid. (**b1**–**d1**) Shows three different conic-droppers of angle 16.9°, 31.9° (Supplementary Video 1) and 58.3° (Supplementary Video 2) respectively. **(b2**) A dropper with slope 16.9° extracting a small amount of PDMS at the tip. (**c2**) and (**d2**) are droppers with slope 31.9° and 58.3° respectively that extracts a fixed volume of PDMS, a droplet hangs over the tip of each of cone dropper. (**b3**) A holder (1 mm thick) captures no droplet as cone dropper in (**b2**) did not dispense any droplets. (**c3**) and (**d3**) both collects a droplet from by the cone-droppers in (**c2**) and (**d2**). However, the droplet held in (**d2**) fails to capture the first droplet (supplementary video 3). Red dotted line indicates the angle, white dotted lines shows the position of the holder. All the images are on a scale bar of 1 mm.

In the passive process, we need to provide the equivalent of a negative and positive pressure in active dispense in order to extract and dispense single droplets. The proposed study is separated into three parts; immersion/extract, dispensing and capture. We chose liquid silicone polymer (polydimethylsiloxane (PDMS)) as it have high viscosity that can be shaped in room temperature and cured under low heat (70–200 °C). The cured PDMS lenses exhibit high refractive indices (n = 1.39–1.55) and optical transparency (>95%) within visible spectrum of the wavelength, without encountering issues of nominal aging (yellowing), and are resilient to high temperatures (>125 °C). We first devise a cone-shaped substrate that is immersed into a basin of pre-cured silicone polymer solution. The shape of the cone is designed to promote capillary action as it is being immersed and pulled away from the basin as shown in Fig. 1. Immediately after immersion, Fig. 1(a1), the extracting action, shown in Fig. 1(a2), exhibit a clear meniscus and capillary forces sufficient to extract a small amount of liquid passively from the basin. The shape of cone is designed to provide adequate surface area for sufficient capillary forces (>1 mm height) to extract a certain amount of liquid. Figure 1(b1,c1,d1) shows three 3D printed cone-shaped substrates with an increasing angle of inclination of 16.9°, 39.9° and 58.3° respectively being used to extract the liquid. Figure 1(b2,c2,d2) shows the respective extracted liquid with individual droplets hanging over the cone tip. Each cone is then immersed into a basin of pre-cured PDMS for short interval (2–3 seconds) before it is extracted, as depicted through a transparent cuvette in Fig. 1(a1) (immersion) and 1(a2) (extract). After extraction, a droplet is left hanging over the tip of the cones as shown in Fig. 1(b2–d2). At every dip, the cone design extracts a fixed amount of PDMS which will then elongate to form a single primary droplet. However, this technique relies on the immersion level of each dropper which can influence the amount of droplet being extracted.

In the second part of our study, we studied the effect of cone angle and the dynamics of the droplet formed after immersion. The reason is to ensure controlled dispensing of a single drop of silicone fluid. The primary motivation here is to overcome the capillary forces used in the immersion process. If the capillary forces is stronger than the liquid mass, the extracted volume does not flow and therefore cannot be dispensed. Hence, there is a need to have sufficient instability that forces the liquid passively flow towards a holder where a dominant gravitational force preside over the extracted droplet to create the dripping process^24^. We can also associate the cone design to the Bond number of the fluid, 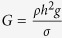, where *σ* is the surface tension, *ρ* is the density, *g* is the gravitational acceleration and *h* is the height of the liquid droplet^25^. In order for the cone to dispense a droplet, it needs to possess a larger *G* value ≫ 1. Based on the fluidic properties of PDMS (*σ* = 21 dyne/cm, *ρ* = 0.97 g/cm^3^, and *g* = 980 cm/s^2^), we observed that an increase in the height of the droplet (*h*) leads to an increase of the Bond number (G ≫ 1), which leads to liquid instability and initiates formation of a discrete droplet. Next we examine the droplet hanging off each of the conical-dropper. The height of each hanging droplet are shown to vary from 0.5 mm, 2.2 mm and 5.5 mm (dotted white lines in Fig. 1(b2),(c2) and (d2) from cone angles 16.9°, 39.9° and 58.3° respectively. This translates to a Bond number of 0.11, 2.19 and 13.69 respectively. As expected, our experiment confirmed, shown in Fig. 1(b2) and (b3), that a shallower cone extracts sufficient fluid to form a single hanging droplet via capillary forces but fails to be transferred onto the holder as shown in Fig. 1 (b3). With cone angle larger than 39.1°, a larger droplet extracted that reliably forms a single droplet (supplementary video 1) that is then captured by holder by the holder as shown in Fig. 1(c3). As the cone angle reaches 58.3° as shown in Fig. 1(c1), we observed that a large amount of liquid is extracted that leads to several successive droplets (supplementary video 2). This is reflected the unsuccessful deposition of the droplet onto the holder. The increase of extracted volume can be attributed to increase capillary action using a steeper cone. Since during immersion, the increase in the cone angle is likely to give rise to an increased liquid height over the cone.

In the process of capturing individual droplet, we uncovered that the distance between the holder and tip of the cone can play an important role. This is because the holder need to be able to capture each droplet while allowing sufficient distance for fluid to elongate and detach into a droplet. Based on Rayleigh-Plateau instability, a droplet formation can be characterised by a sinusoidal modulation where the optimal wavelength *λ* of the modulation is proportional to the flowing jet, r by 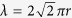
26. Through several empirical experiments we show that when a holder is kept at a distance of *λ*_*opt*_/6 ≈ 0.5 mm, where radius of the jet, *r* = 0.34 mm, s the extracted liquid can be directly transferred onto the holder as a single discrete droplet. If the holder is placed at distance < (*λ*_*opt*_/8), we observed that the droplet is more likely to fall through the holder as shown in Fig. 1d3 (supplementary video 3).

**Figure 2:**
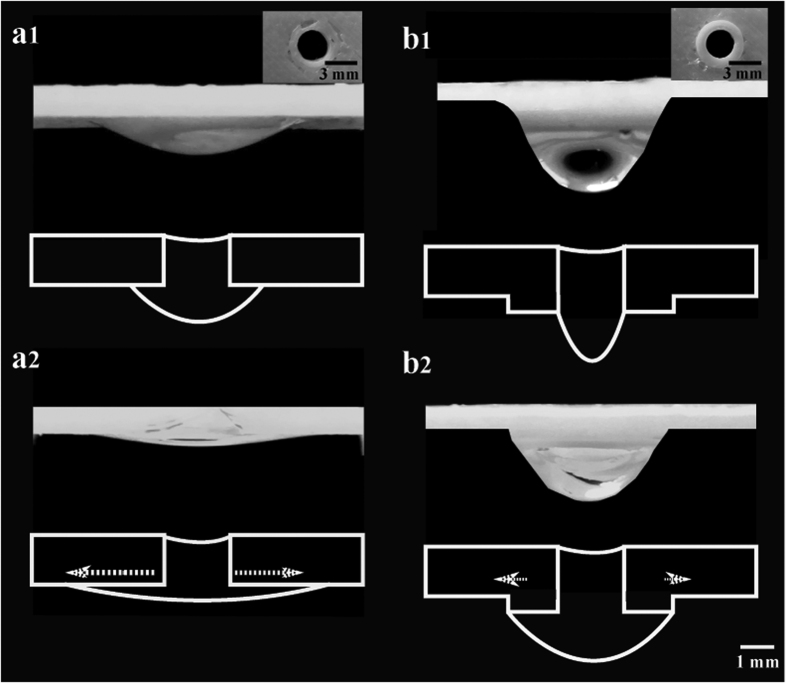
Reduced droplet wetting via pinning on the holder using 3D printed barrier. (**a1**) A droplet first deposited onto a holder without a barrier (inset (**a1**)). (**a2**) Droplet with reduced curvatures as it spread across the substrate. (**b1**) Shows a droplet first deposited onto a holder with a fixed barrier (height of 1.04 mm) (Supplementary Video 4). (**b2**) Shows droplet holding its shape in the presence of the barrier showing the effect of liquid pinning. (Supplementary Video 5). The holder images are on a scale bar of 1 mm.

Finally, once the drop is formed onto the holder, the droplet needs to retains its pendant shape^27^ during curing so as to obtain an optimal convex lens. This process relies on balancing capillary forces with large gravitational forces and also considerable wetting action on the surface of the holder. As a start, we use a flat substrate with a through-hole, where the thickness of the holder creates a fixed capillary force to hold the droplet in position. The appropriate thickness is approximated to the capillary length of liquid silicone (~1.5 mm). Figure 2(a1) and (b1) shows the proposed substrate where a through-hole with a thickness was chosen to be ~1–1.2 mm. However, as liquid comes into contact with a solid interface, there will be considerable wetting^28^. Figure 2 demonstrates the impact of wetting after dispensing. By just relying on the through-hole, as shown in Fig. 2a (Supplementary Video 4), the deposited fluid assumes a pancake shape that quickly disperses, resulting in a long focal length lens each time. Hence, it fails to retain the shape of the droplet. To mitigate the process of wetting, we incorporate a small sharp circular edge, Fig. 2(b1) inset, imposes physical liquid pinning effects as shown in Fig. 2(b1) and b2. Since wetting only occurs over a continuous surface, the edge serves as a barrier so that the liquid stays pinned at the circular edge^29^ as shown in supplementary video 4 and 5 shows how the curvature of the pendant drop with and without that the barrier respectively. The curvature of the droplet generated is pinned (self-aligned^29^ s) using the barrier and so retain the ideal convex shape of a lens (Supplementary Videos 4 and 5).

**Figure 3:**
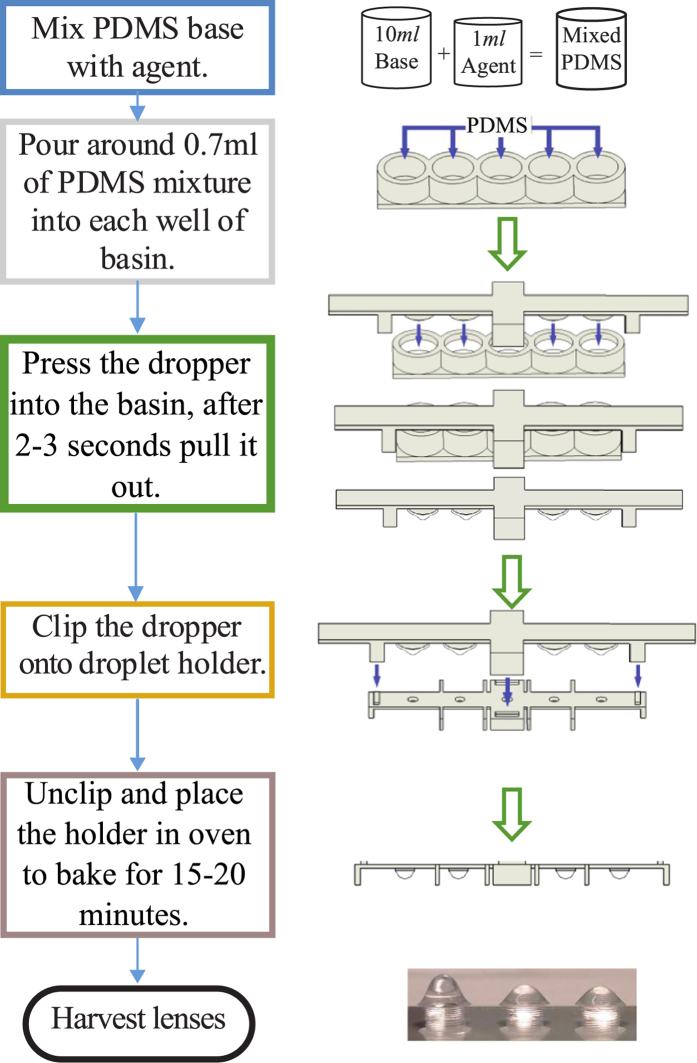
A flowchart depicting the passive droplet dispenser lens-making process supported by schematics. The lens making protocol has been elaborately in Supplementary Information S1 and S2.

To sum up this section, we devised a simple workflow/guide for users to print and use, as shown in Fig. 3. The three parts are designed using Solidworks© (design files provided as supplementary information S1) and printed using a 3D-UP Mini printer (process protocol provided as supplementary information S3), which are: (i) the basins, to hold the PDMS mixture, (ii) the droplet-holders with holes to hold the droplets and (iii) the droppers that are used to extract PDMS mixture and allow the droplets to be formed and dripped onto the holder (shown in Supplementary Information S2). Briefly, we discuss the list of the steps. Firstly, the PDMS base agent is mixed with the curing agent with a ratio of 10:1 for approximately 3–4 minutes. After which, the solution is degassed (60 minutes - desiccator, 20–30 minutes - vacuum pump) to remove air bubbles. A regular 3 ml plastic syringe then extracts a small amount of PDMS (~0.7 ml) that fills each basin (i). The droppers are then pressed into the holes of the basin to extract PDMS droplets as shown in Fig. 3. The droppers are placed on the droplet-holder using a mechanical clip (as shown in Supplementary Information S2) which is used to ensure that each dropper is aligned and positioned at a desired distance over each hole and a misalignment of up to ~10% will lead to successful transfer of the droplet. Depending on the viscosity of the mixed PDMS, it takes about 15–20 seconds for the droplets to be dispensed onto the droplet holder. Once the droplets are formed on the droplet-holder, the holder is placed in the oven at 70 °C for 15–20 minutes. After curing, the lenses are then removed from the droplet holder. The 3D printed kits (Supplementary Information S1 and S2) can be re-used. For completeness, we also investigate the impact of increased capillary forces in the droplet holder by increase its thickness to 2.8 mm. Here, the lenses cured shows a thick base due to increased capillary force (shown in Supplementary Information S4). To this end, we showed that the appropriate combination of the cone angle, lens holder distance and holder design greatly affects how the convexity of silicone droplets can be passively held in position.

Although this marks a significant advancement of the passive dispenser approach over previously published works is the high throughput process by using basic tools that can be easily 3D printed, the quality of this form of lenses needs to be further verified. In the next section, the optical properties of the silicone lenses are carefully characterised with a wavefront sensor (Shack-Hartmann wavefront sensor) and an optical surface profiler (white light interferometer).

## Results

### Optical quality, focal length and imaging resolution

**Figure 4:**
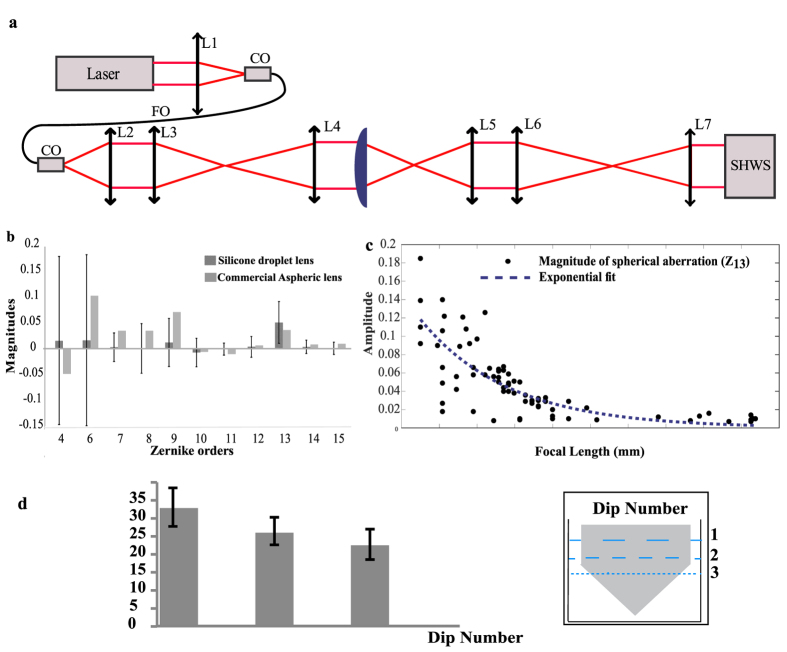
Optical aberrations and focal lengths of silicone lenses. (**a**) The optical bench setup using a SHWS that has been used for optical characterization of the lenses. (**b**) Optical characteristics of the lenses are summarized using Zernike modes of various orders. Comparison among the silicone lenses and a commercial aspheric lens has been shown in a chart. Zernike modes 1, 2, 3 and 5 which are piston, tilt and tip, were not included as they do not represent true aberrations from the lenses. Higher Zernike modes with negligible values were also ignored. (**c**) Plot of magnitude (unit wavelengths) of spherical aberrations (Zernike mode 13) with lenses of various focal lengths. (**d**) Relationship between the dip numbers and the corresponding focal lengths of the lenses.

A series of optical (wavefront) and surface profile measurements are conducted so as to compare the quality of the droplet lenses against an existing off-the-shelf lens. The wavefront measurements quantify the magnitude of each optical aberrations based on Zernike modes. The surface profiler maps out the surface roughness of the lenses with nanometer precision. The Shack-Hartmann Wavefront Sensor (SHWS) (Thorlabs, WFS150–5C) measures and decomposes optical aberrations into a set of orthonormal series of Zernike polynomials using an array of micro-lenses and a charged coupled device (CCD). Figure 4a shows a schematic of the SHWS setup used in the wavefront measurement. A coherent laser diode (OBIS-Coherent, wavelength = 660 nm) is used as the light source to test the aberrations. The first lens L_1_ couples a low divergence output laser beam into a single mode fibre. The output from the single mode fibre is then collimated with L_2_. The arrangements of lenses (L_3_– L_7_) are chosen to ensure matching conjugate planes and beam waist onto the input face of the SHWS. The output beam goes through a pair of lenses (L_3_, L_4_) to match the diameter (~2 mm) of the sample lens (PDMS or commercial aspheric). The sample lens under test is placed between L_4_ and L_5_, where L_5_ collimates the output beam. The diameter of the beam output of L_5_ is ~20 mm. Another pair of lenses (L_6_ and L_7_) is used to reduce the beam diameter size to fill active area of the SHWS. The wavefront Zernike polynomials were used to express the magnitudes of the optical aberrations present in the lenses. Figure 4b shows the mean distribution of magnitude of the aberrations of over 75 PDMS lenses and an off-the-shelf aspheric lens (Thorlabs A110). The results show that astigmatism and spherical aberrations from the silicone lenses are significantly larger than those from aspheric lenses. Astigmatism has most likely been caused by imperfect print of the second substrate and unstable positioning during curing whereas spherical aberration has been caused by the non-aplanatic curvatures of the silicone lenses which are consequences of the almost conical shape of the curvatures. This effect is consistent with lenses with shorter focal lengths where the spherical aberration is shown to increase, as illustrated in Fig. 4c. In principle, the extraction procedure relies on passive extraction must provide a very stable control over the volume of each extracted droplet. However, the immersion and extraction process is perform manually which give rises to a range of droplet size and therefore focal length. This expected diversity is then characterised based on dip number as illustrated in Fig. 4d where the action of each dip produces lenses with increasing focal lengths ranging from ~3.5 mm to ~15 mm as less liquid silicone is being extracted.

**Figure 5:**
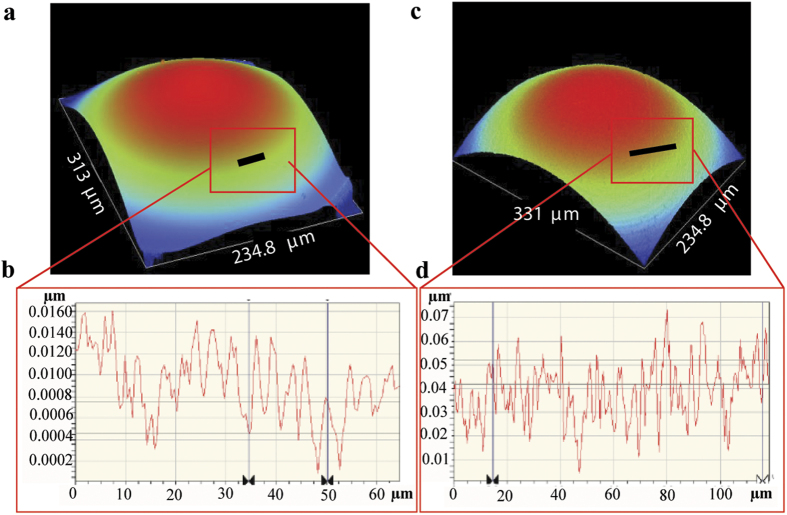
Optical profiling of surface roughness of a silicone lens and a commercial lens. (**a**) 3D map of curvature of front surface of a silicone lens. (**b**) A cross-section (line plot) of the surface, measuring the surface roughness of the silicone lens. (**c**) 3D map of the curvature of the front surface of a commercial aspheric lens. (**d**) Corresponding line plot of cross section of the surface.

During curing, it is also possible that the delicate liquid air interface of droplet formation can introduce uneven surfaces on the silicone droplets^30^. To study that surface roughness of the lenses were measured using an optical profiler (Wyko NT9100: Mirau interferometer) and compared against commercial aspheric lenses. The results, as shown in Fig. 5a and b, in the form of the 3D maps and a cross-sectional plot, suggest that the silicone lenses have a highly conforming surface. Commercial aspheric lenses are shown in Fig. 5c and d. The measurements suggest that the silicone lenses possess lower magnitude of surface roughness compared to the commercial aspheric lens. The RMS value of the surface roughness of the commercial aspheric lens was found to be 15 nm and for the silicone lenses harvested using the passive dispenser was found to be 3.21 nm for the front surface and 1.91 nm for the back surface.

**Figure 6:**
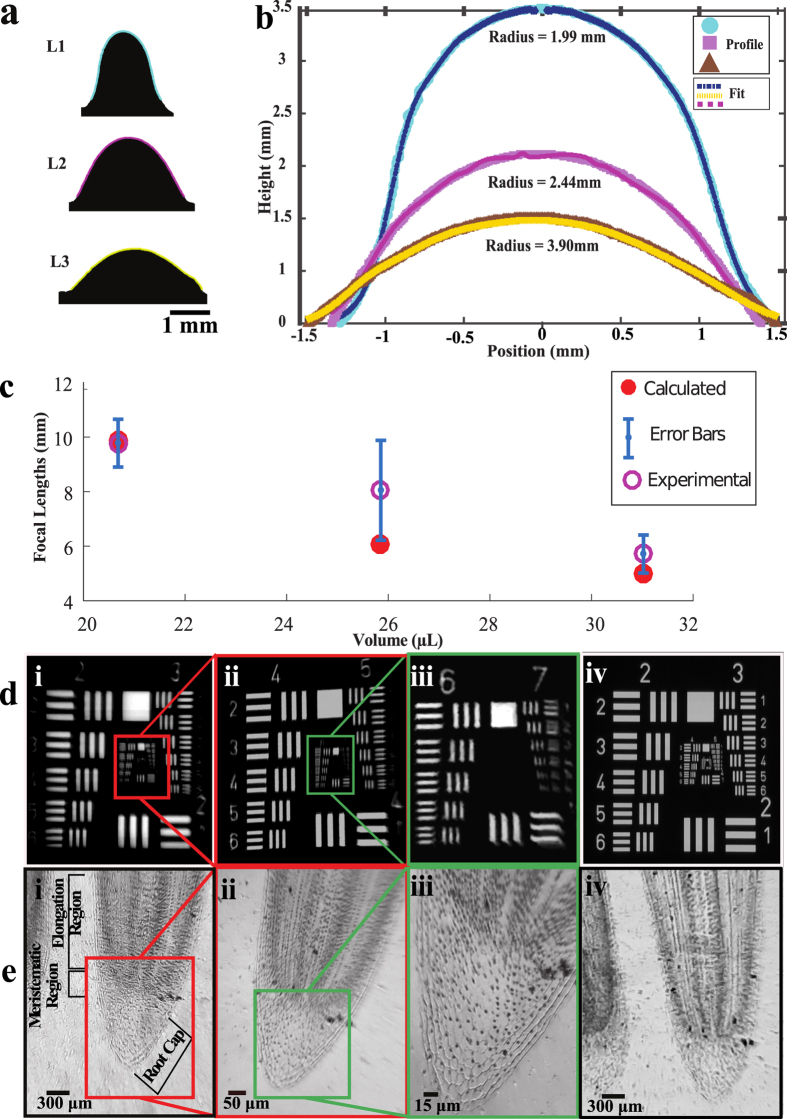
Lens curvature profiling and imaging. (**a**) Curve tracing of the front surface of three silicone lenses fabricated using the passive dispenser. (**b**) Gaussian fitting of the corresponding profiles and the calculated radii of curvatures. (**c**) Measured and calculated focal lengths plotted as a function of the volume of the harvested lenses. (**d1**–**d4**) Magnified images of the USAF target card of magnifications 30X, 50X, 80X and 25X respectively. (**e1**–**e4**) Images of an onion root tip at different magnifications.

Overall, the extraction procedure relies on passive extraction which should in principle, created a very stable control over each droplet. However, in our experiment, the liquid extraction process is manually carried out which will give rise to a range of focal length of lenses, as shown previously in Fig. 4d. As such, it is important to quantify the focal length of the lenses. While standard focal length measurement provides an accurate measure of the focal length, it often requires a complex imaging setup. Here, we propose a simpler method to quantify the focal lengths by capturing the profiles of the lenses and then mapping out the radii of the curvatures. The focal lengths are then compared with the standard focal length measurements for accuracy. Figure 6a shows the outline of 3 silicone lenses that were captured using a simple optical setup consisting of a spherical lens of 30 mm focal length, Raspberry Pi camera and illuminating light. Figure 6b shows the surface profile of different silicone lenses. The surfaces of the lenses were found to be parabolic with varying heights and curvatures. All of the profiles were captured through simple edge detection technique and then fitted onto a Gaussian fit (shown in Fig. 6b). The focal lengths were calculated at the vertex of the curvature of the lenses. Silicone lenses with longer focal length lenses have a smaller amount of PDMS that result in a reduction of radii of curvatures of the lenses and vice versa. Based on the curvature profiles shown in Fig. 6a,b, the focal lengths have been calculated using modified lens maker equation 
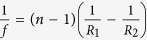
, where *n* is the refractive index of the lens and *R*_*1*_ and *R*_*2*_ are the radii of curvatures of each lens, assuming *R*_2_ = ∞. Figure 6c summarized both the measured and calculated focal lengths of the lenses over volume of dispensed PDMS. It is nominally expected that a higher volume results in a higher degree of convexity due to higher sag and thus have shorter focal lengths. We also observed additional fit to measure the asphericity of the lenses using the aspheric curve formula^31^, 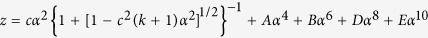, where *z* is the sag, *α* is the radial distance from the optical axis, *c* is the curvature (/mm), *k* is the conic constant and *A, B, D* and *E* are higher order aspheric coefficients (summarized in Table 1). Based on the calculated focal lengths of the two fitting approaches, it has been observed that there is a closer correlation of the measured focal length with the simple nonlinear fit than the aspheric lenses. This indicates that the finite clear aperture of lenses is limited to a small region around the central portion of lenses like a spherical lens. Figure 6(d1–d3) shows images of an USAF 1951 card imaged using different silicone lenses of different magnifications and a commercial aspheric glass lens. In Figure 6(d1) groups 2 and 3 of the target card are clearly visible with up to element 4 of group 3 resolvable (resulting into a resolution of ~44 μm). As seen in Fig. 6(d2) groups 4 and 5 can be resolved up to element 6, with ~8.77 μm resolution. The lens with the longest curvature produces an image in Fig. 6(d3) can resolve up to element 2 of group 7 yielding a resolution of ~3.4 μm. Figure 6(e1) to (e3) show images of a histological slide of onion cells at the tip of an onion root imaged using the same lenses as the USAF target card has been imaged. The lowest resolution image shown in Fig. 6(e1) shows the wider field-of-view structure of the onion root tip where the root cap and meristematic region are seen with a part of elongation region. In Figure 6(e2) the onion root image shows the cell walls and cells resolvable to less than 15 μm. In Figure 6(d4) and (e4) shows images of the USAF target card and the tip of onion root has been captured using a commercial aspheric lens of focal length 18.1 mm. The performance is comparable with the silicone lenses. The overall magnifications (optical and digital) of the system for the different lenses based on the images are as follows; Fig. 6 d1, e1 are 30X, Fig. 6 d2, e2 are 50X, Fig. 6 d3, e3 are 80X and Fig. 6 d4, e4 are 25X.

### Portable high resolution imaging platform

**Figure 7:**
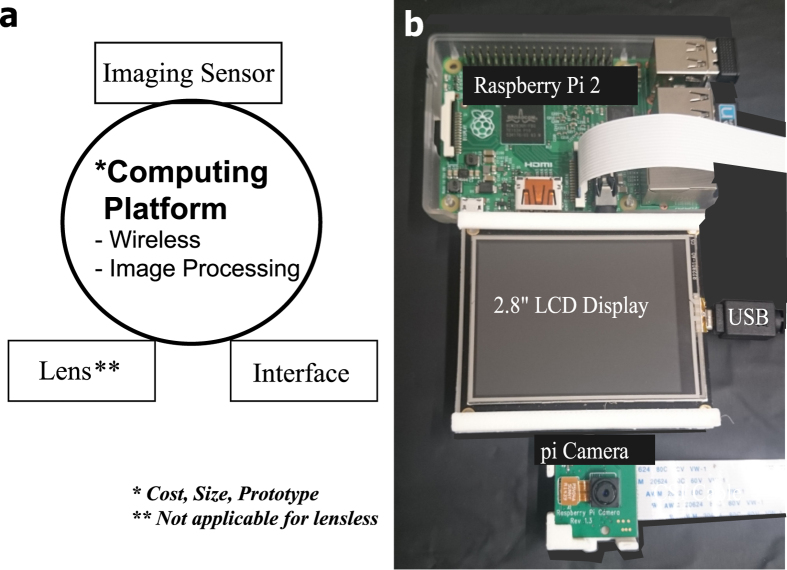
Overview of a portable imaging system. (**a**) Schematic representing the components required for a compact microscopic imaging system. (**b**) A Raspberry Pi -2, the Pi camera and a 2.8″ LCD display is another option for designing a small imaging system.

**Figure 8:**
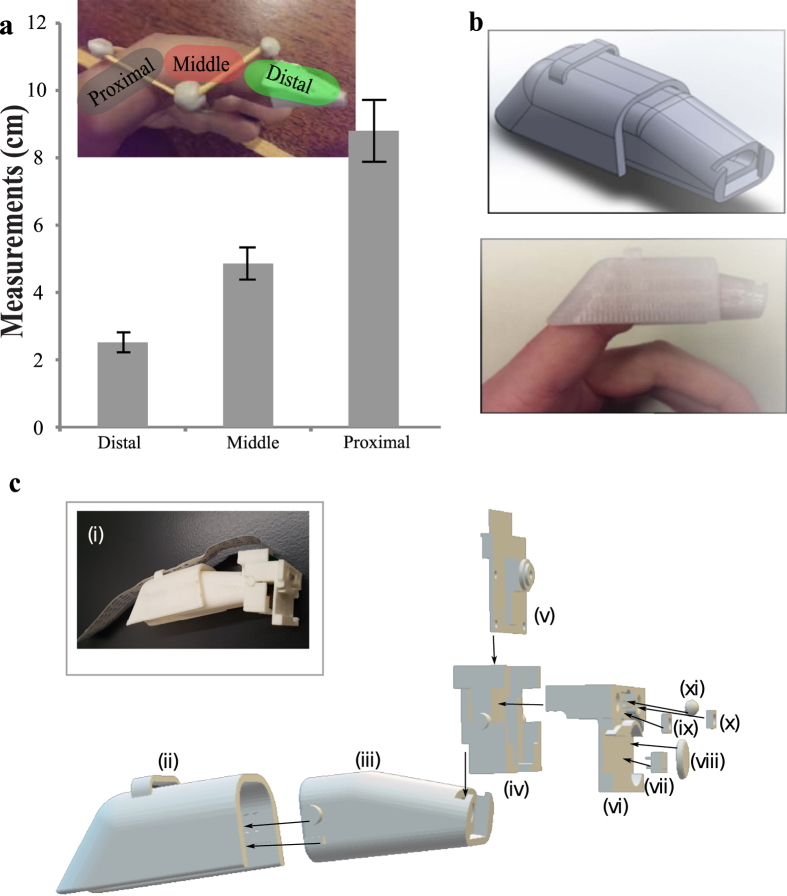
Portable thimble imaging system. (**a**) Chart showing average of length of index fingers (n = 5). (**b**) (top) 3D designs of the thimble mount (bottom) thimble mounted on the index finger. (**c**) Thimble design. (i) Completed 3D printed thimble mount. (ii) Back section of the finger brace. (iii) Front section of the finger brace. (iv) Back connector of camera holder. (v) 3D schematic of Raspberry Pi camera. (vi) Front part of the camera holder. (vii) DIP switch to control illumination. (viii) Coin cell battery to supply power for the light emitting diodes. (ix–x) Two neopixel white LEDs. (xi) A silicone lens, designed using the passive dispenser, aligned with the Raspberry Pi camera.

For lens based microscopes, the components include magnifying miniature lenses, an imaging sensor, and a stand-alone computing platform with interface (hardware and software) as shown in Fig. 7a. Other possible choices of miniature imaging systems that have been considered that include Naneye is an endoscope-based camera unit that is a size of around 1 × 1 mm, ideal for miniature systems and has been used in the portable imaging system called the Magic Finger project^32^. The approximate cost of the camera and evaluation board is ~US$1550 for a very modest resolution of 250 × 250 pixels. In addition, the imaging unit does not have an on-board computing system for image capturing and image processing. Alternatively, it is always possible to use a conventional computer internet camera (webcam) with a generic image processing board IMX6 to capture images and standard large screen display. In contrast, Raspberry Pi, commonly billed as credit card sized computer, is a highly accessible and affordable computing platform targeted to deliver cost efficiency, compact design and the ability to tailor multiple functions to the imaging requirement of a compact imaging system, as depicted in Fig. 7b, at a very modest price of ~US$77. As compared with smartphone imaging system, the Raspberry Pi system offers a greater degree of flexibility because the display, camera and computing unit are designed as individual interconnecting modules. To illustrate the portability and modular properties of imaging system, we move to develop a wearable imaging system attachment; a thimble camera device. The thimble attachment is designed for the index finger much like the Magic Finger system^32^. Instead of using soft straps, here, a multi-part 3D printed brace is designed to attach a high resolution camera at the fingertip and a small portable display is connected to deliver immediate visual feedback. The dimension of the thimble finger-brace are based on measurement of length of index fingers from different volunteers (n = 5). A summary of the measurements is articulated in Fig. 8a. A two-part thimble brace, as shown in Fig. 8b and c, is used to accommodate internal and external fillets with the back and front part of the finger brace. The two sections of the finger brace slide along each other so that the brace can be secured onto different users with different lengths in the middle and proximal phalanx. The front section of the thimble was also designed to house a Raspberry Pi camera, as shown in Fig. 8c(i) 3D printed- inset, and 3D schematic Fig. 8c(ii–xi).

**Figure 9:**
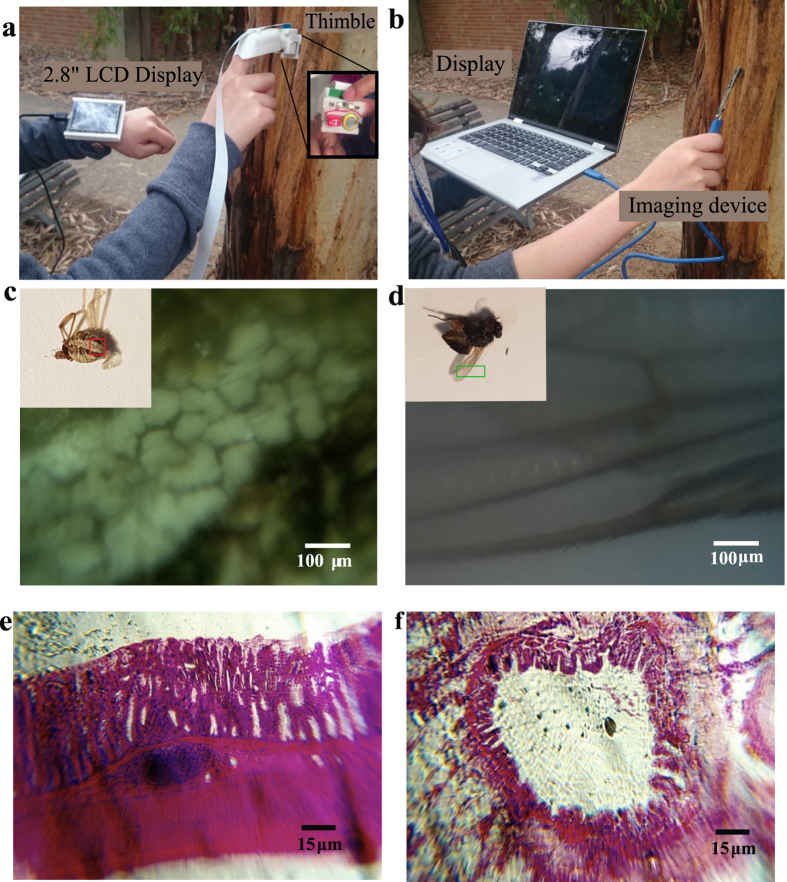
Thimble imaging device with macroscopic and microscopic imaging. (**a**) Handheld imaging device used with standard laptop for imaging outdoors. (**b**) Thimble imaging system with miniature LCD display in an outdoor environment. (**c**) A macroscopic image of a section of the back of a *Latrodectus hasseltii* (male redback spider of around 3 mm body length) has been captured using the thimble imaging device. (**d**) Macroscopic image of section of a wing of a regular *Musca domestica* (house fly) has been captured using the thimble imaging device. Microscopic imaging of histology slides of (**e**) Human colon and (**f**) Liver tissue.

Next we demonstrate the portability of the new system when including additional hardware i.e. computer and display. As shown in Fig. 9a, a small LCD display worn on a wristband is directly attached to the credit card sized Raspberry Pi unit (shown out of view here). As compared with a conventional hand-held lower resolution imaging device (webcam) as shown in Fig. 9b, the thimble imaging system is significantly smaller. Furthermore, in the thimble design, both hands are essentially free to handle additional objects that are otherwise immobilised by a traditional laptop mobile camera. This design was found to be helpful when imaging small insects hidden underneath the leaves or other obstructed samples which otherwise would be challenging to reach with traditional microscopes, or even with smartphone based microscopes where the position of display and camera are fixed. We further demonstrate the macroscopic imaging capability of the new thimble imaging system. Figure 9c and d show macroscale images of a segment of the back of a *Latrodectus hasseltii* (inset - male redback spider of around 3 mm body length) and section of a wing of a regular *Musca domestica* (inset - house fly) imaged with the thimble imaging system. In addition to macroscale imaging, the camera, mounted on a fixed micrometer stage, is also used for microscopic imaging of known histological slides in Fig. 9e human colon and Fig. 9f liver tissue.

### Discussion

Recent combination of consumer mobile imaging system and disposable lenses have ushered in practical *in-vivo* imaging screening on mobile devices^10^,^18^,^33^ for primary care and low resource settings. We successfully implemented the use of a passive droplet dispenser that provides a direct and low-cost alternative to fabricating lenses. Our passive approach is designed by carefully considering interaction of fluidic forces such as surface tension, capillary forces, gravitational force and fluid dripping process. While there is significant variability among the focal lengths of the lenses, the simplicity of the approach makes this a viable approach for rapid production of high quality short focal length lenses for high resolution imaging. The complexity of this approach is significantly reduced compared to a syringe-like dispenser. Moreover, the scalability is high since the lens fabrication tools can be easily optimised via 3D printed and also parallelized to fabricate multiple lenses simultaneously. For active dispensing, the size of macrodroplets will need to be controlled by using various pumps and nozzle diameter adding complexity. Finally, we showcase a modular microscope design based on Raspberry Pi system on a thimble imaging setup. It removes rigid design (fixed arrangement of camera and display) of existing consumer smartphone systems and offers much higher configurability and portability. The new design closely resembles a digital endoscope or inspection scopes albeit it uses the finger as an actuator to manipulate the camera. A main drawback of the silicone lens is the significant optical aberrations that become apparent at the peripheral of the lenses, see Fig. 7e and f. While, it is possible to redesign the fabrication step to yield high quality lenses, it will require an entirely new process^17^. Instead, we proposed the next step is to use the computational power of the Raspberry Pi to implement a computational imaging technique such as Fourier Ptychography^34^ so as to remove the optical aberrations using iterative phase retrieval. The combination of both the silicone lenses and computational image processing will greatly enhance the capabilities of the portable microscope for capturing images over large field of view at high resolution.

## Additional Information

**How to cite this article**: Kamal, T. *et al*. Design and fabrication of a passive droplet dispenser for portable high resolution imaging system. *Sci. Rep.* **7**, 41482; doi: 10.1038/srep41482 (2017).

**Publisher's note:** Springer Nature remains neutral with regard to jurisdictional claims in published maps and institutional affiliations.

## Supplementary Material

Supplementary Video 1

Supplementary Video 2

Supplementary Video 3

Supplementary Video 4

Supplementary Video 5

Supplementary Information

## Figures and Tables

**Table 1:** Aspheric Curve fitting data for different lens curvature profiles.

	Lens 1 (L1)	Lens 2 (L2)	Lens 3 (L3)
Curvature (/mm)	−1.189	−0.7106	−0.5236
Conic constant	−0.6	−0.75	−0.9
RMSE	0.1830	0.0179	0.0234
Aspheric Coefficients			
A	0.4031	−0.7892	−0.1441
B	−6.46	0.9704	0.122
D	6.946	−0.8609	0.004547
E	−2.044	0.3115	−0.01959
