# Supplementary material for: Design and fabrication of a passive droplet dispenser for portable high resolution imaging system
Source: Sci Rep. 2017 Jan 27;7:41482. doi: 10.1038/srep41482 (PMC5269729; doi:10.1038/srep41482)
Supplement: Supplementary Information [file srep41482-s6.pdf]

Design and fabrication of a passive droplet dispenser for portable high resolution imaging system

Tahseen Kamal[1], Rachel Watkins[1], Zijian Cen[1], Jaden Rubinstein[1], Gary Kong[2] and W. M. Lee[1*]

[1]*Research School of Engineering, College of Engineering and Computer Science, The Australian National University, North Road, ACT 2601, Australia.*

[2]*Plant Biosecurity Cooperative Research Centre, LPO Box 5012, Bruce, ACT 2617.*

*****Corresponding Author: steve.lee@anu.edu.au.

Supplementary information (S1):

The STereoLithography (STL) design files of the passive droplet dispenser can be found from the following links.

1. Basins.
2. Droppers.
3. Droplet-holders.

Supplementary Information (S2):

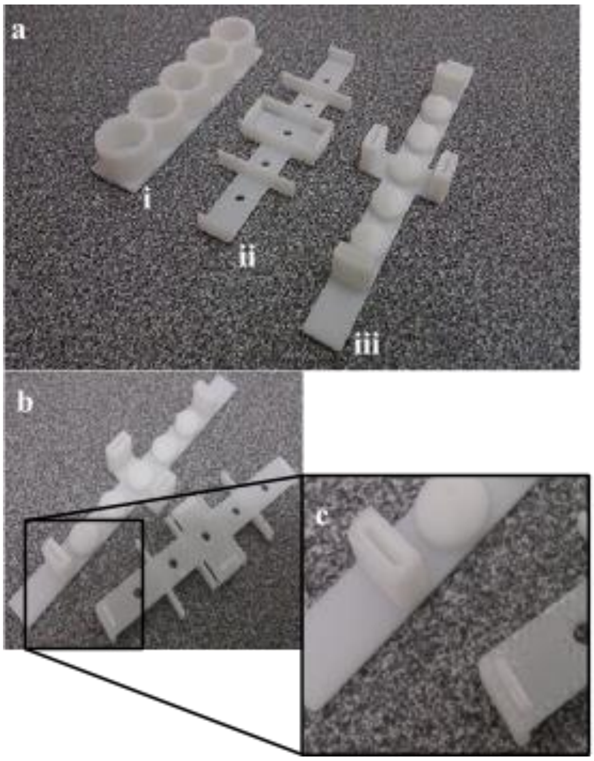

Figure 1: The passive dispensing 3D substrates. (a) Different parts of the passive dispenser. (i) Basins, (ii) Droplet-holder, (iii) Conic droppers. (b)-(c) Mechanical clip attachment to clip the dropper onto the holder which ensures proper alignment.

Supplementary information (S3):

The protocol for using the passive droplet dispenser has been developed here. In this section we have outlined the necessary skills, time, tools, associated risks and the process flow diagrams of the 3D printing and silicone lens making processes.

1. **Necessary Skills:**

   The passive droplet dispensing process is a simple and easy to adapt approach to acquire dripping-drops of Polydimethylsiloxane (PDMS). The skills required to follow the procedure are,

   - Basic idea of 3D printing which can be acquired by following the attached 3D printing process diagram.
   - Basic knowledge about measurements.

- Basic idea of using an oven of temperature around 70°C.
- Basic idea of using spatula with sharp edge to extract the 3D printed parts from the printing platform.

2. Time required:

To complete the 3D printing process the required time is approximately 2 hours and 30 minutes using an Up Mini 3D printer. An additional time of 15 minutes should be considered to preheat the printing platform.

For the PDMS droplets to work the PDMS base and curing agent have to be pre-mixed with a ratio of 10:1. It takes about 60 minutes to degas 20 ml of mixed PDMS using a desiccator. This time depends on the amount of mixture also. In the event of availability of a vacuum pump the time required to degas the mixture can be 20 minutes for 20 ml of PDMS.

Once the 3D printing substrates and the mixture are ready, it takes about 2-3 minutes to extract droplets onto the droplet-holder using the substrates.

Finally in an oven preheat at 70°C, it takes around 20 minutes of baking to get the cured PDMS lenses. Alternatively, the droplet-holder can be left in room temperature for 48 hours for the PDMS to be cured completely. In this case, caution should be taken to keep the droplets covered to prevent dust accumulation which will deteriorate the quality of the lenses.

3. Tools needed:

To complete the 3D printing process the following tools are needed to be used:

- A 3D printer (Up mini).
- ABS filament (Acrylonitrile Butadiene Styrene).
- 3D printer accessories, such as a spatula with sharp edge to extract the parts from the support material and the printing platform.
- Pair of gloves for safety.

To complete the passive dispenser lens making process the following tools are required:

- The 3D printed passive dispenser parts, basins, droppers and droplet-holders.
- PDMS, Sylgard 184, comes in two parts as base and curing agent.
- An oven, FALC Instruments.
- A desiccator.
- A cup to mix the PDMS.
- A spoon to stir the PDMS base with the curing agent.
- Appropriate scales to measure the PDMS base and the curing agent.
- A 3 ml syringe to extract PDMS.
- A pair of tweezers.

4. The process flow diagrams:
In Figure 1 the process flow diagrams for 3D printing and silicone lens-making are illustrated.

a. 3D printing process diagram.
b. Silicone lens making process diagram using passive dispenser.

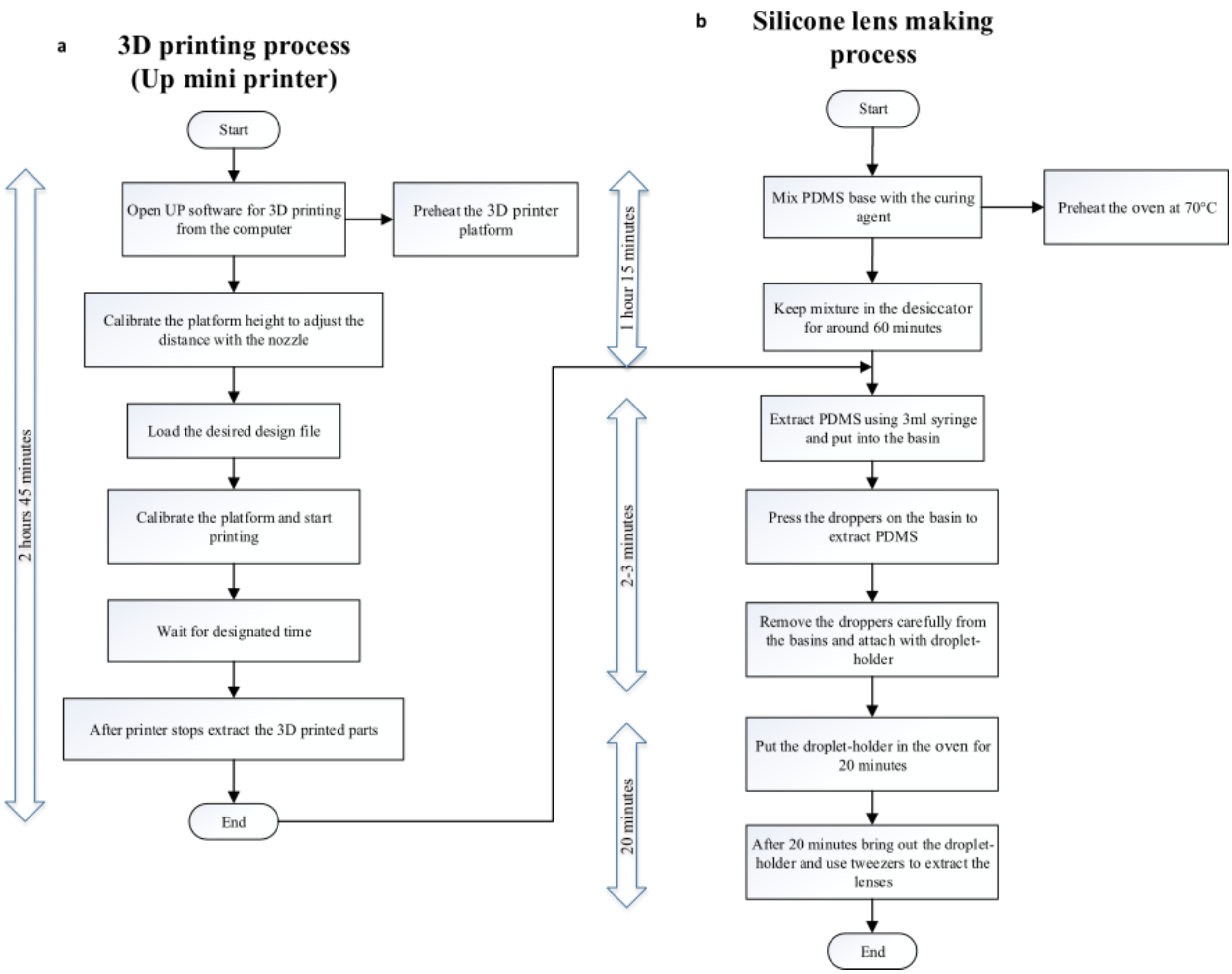

Figure 2: Process flow diagrams for the 3D printing process and lens-making process.

Supplementary Information (S4):

Thick Holder Performance:

The thick holder provides higher capillary force upwards so the curvature of the harvested lens is smaller than desired. Also the thicker stem at the base of the lens makes it a thick lens which is not desired from our design goals.

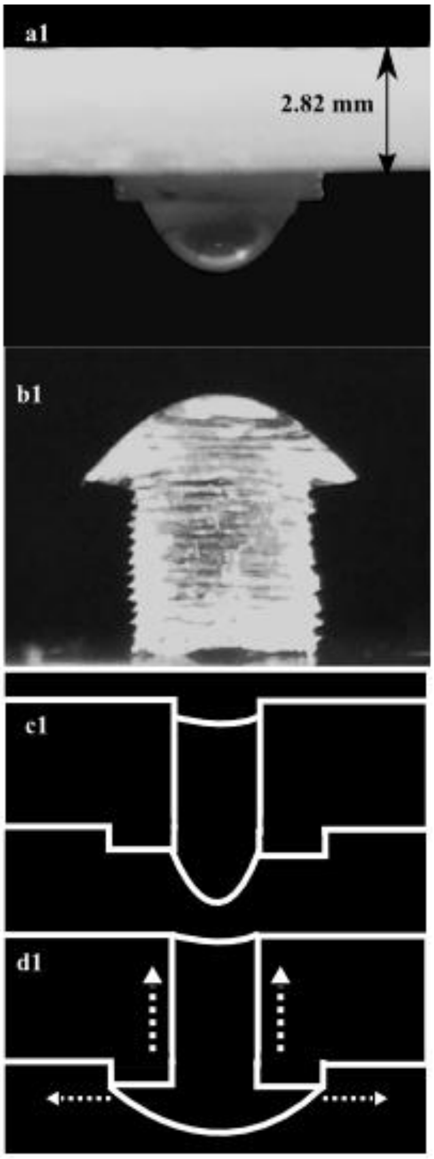

Figure 3: A droplet-holder of 2.8 mm thickness harvesting a droplet lens with small curvature and a long stem at the base.

Wavelength comparison:

The following figure shows fluid filaments generated by two different cone-shaped droppers. Based on the measured values of wavelengths, the distance between the tip of the cone to the holder has been chosen as a factor of λm/6, where λm is the wavelength generated by the proposed dropper.

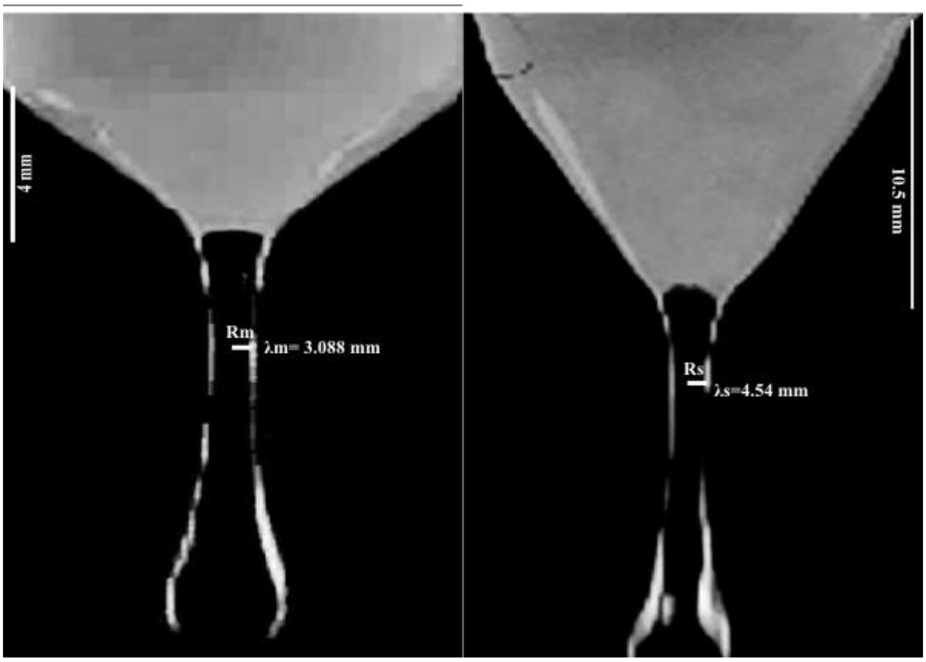

Figure 4: Fluid filament generated by different cone shaped droppers showing that the wavelengths will be 3.088 mm and 4.54 mm respectively for the different droppers. Based on the measurements the distance between the tip of the cone and the holder has been chosen to be ~λm/6 = 0.5 mm , .

Supplementary Video:

The following videos demonstrate a comparison between two different conic substrates. The steeper conic substrate disperses subsequent droplets which essentially is not retained by the holder due to insufficient capillary forces compared to the gravitational forces.

1. Jet flowing from the tip of a conic substrate of slope 31.9°.
2. Jet flowing from the tip of a conic substrate of slope 58.3°.
3. Droplet dripping from the tip of cone of slope 58.3° onto the 1 mm holder.
4. The holder with no barrier showing the PDMS dispersed at the holder due to wetting.
5. The holder with barrier showing the PDMS dispersed droplet pinning.
